# Оценка йодной обеспеченности женщин в первом триместре беременности, проживающих в районах Воронежской области, с различным уровнем потребления йода

**DOI:** 10.14341/probl13646

**Published:** 2025-12-02

**Authors:** А. П. Волынкина, Е. А. Трошина, Н. П. Маколина, О. В. Самофалова, Н. В. Бабий

**Affiliations:** Воронежский государственный медицинский университет им. Н.Н. Бурденко; Центр культуры здоровьяРоссия; Voronezh State Medical University named after N.N. Burdenko; Health Culture CentreRussian Federation; Национальный медицинский исследовательский центр эндокринологии им. академика И.И. ДедоваРоссия; Endocrinology Research CentreRussian Federation; Отдел организации лечебно-профилактической помощи матерям и детям Министерства здравоохранения Воронежской областиРоссия; the Department of Organising Medical and Preventive Care for Mothers and Children of the Ministry of Health of Voronezh OblastRussian Federation; Центр культуры здоровьяРоссия; Health Culture Centre Russian Federation

**Keywords:** женщины, женщины беременные, беременности триместр первый, йод, йода недостаточность, щитовидная железа, тиреоидные гормоны, women, pregnant people, pregnancy trimester first, iodine, iodine deficiency, thyroid gland, thyroid hormones

## Abstract

**АКТУАЛЬНОСТЬ:**

АКТУАЛЬНОСТЬ. В России повсеместно существует риск развития йододефицитных заболеваний (ЙДЗ) из-за недостатка йода в рационе [1, 2]. Основные группы риска, в которых последствия недостатка йода в питании наиболее серьезны, — беременные и кормящие женщины, а также дети от 0 до 3 лет [1, 3, 4].

**ЦЕЛЬ ИССЛЕДОВАНИЯ:**

ЦЕЛЬ ИССЛЕДОВАНИЯ. Оценить йодный статус женщин в первом триместре беременности, проживающих в районах Воронежской области, с различным уровнем потребления йода.

**МАТЕРИАЛЫ И МЕТОДЫ:**

МАТЕРИАЛЫ И МЕТОДЫ. Было обследовано 100 женщин в первом триместре беременности, проживающих в разных районах Воронежской области. Все обследованные заполнили опросник по наличию заболеваний, приему препаратов и питанию, были осмотрены эндокринологом, произведена пальпация ЩЖ, однократно забрана порция дневной мочи (до 12:00) с последующим определением концентрации йода в моче и расчетом медианы йодурии. Проведено исследование образцов пищевой поваренной соли, полученных из домохозяйств, на наличие йода (йодата калия) экспресс-методом качественного анализа. Осуществлен забор крови для определения в сыворотке крови уровня тиреотропного гормона (ТТГ), антител к тиреопероксидазе (AT-TПO), селена, цинка.

**РЕЗУЛЬТАТЫ:**

РЕЗУЛЬТАТЫ. Медианная концентрация йода в моче (мКЙМ) составила 87,35 мкг/л (при нормальном потреблении йода медиана йодурии составляет ≥150–249 мкг/л). Доля использования йодированной соли в домохозяйствах составила 23% (n=23). Только 23% женщин используют йодированную соль, и 6% регулярно принимают йодсодержащие лекарственные препараты, что говорит о недостаточном потреблении йода и практически полном отсутствии йодной профилактики в группе риска развития йододефицитных заболеваний. Медиана уровня ТТГ составила 1,19 мЕд/л, что соответствует референсному интервалу (0,4–4 мЕд/л). Медианная концентрация селена составила 0,098 мкг/мл, что также соответствует референсному интервалу (0,07–0,12 мкг/мл), достоверных различий в концентрации селена среди жителей города Воронежа и районов не установлено.

**ВЫВОД:**

ВЫВОД. Выявлен крайне низкий уровень употребления йодированной соли в домохозяйствах и недостаточное потребление йода у беременных.

## Abstract (en)

**RELEVANCE:**

RELEVANCE. In Russia, there is a widespread risk of developing iodine deficiency disorders (IDD) due to insufficient iodine in the diet [1, 2]. The primary at-risk group, where the consequences of insufficient iodine in the diet are most severe, includes pregnant and breastfeeding women, as well as children aged 0 to 3 years [1, 3, 4].

**THE AIM OF THE STUDY:**

THE AIM OF THE STUDY. To assess the iodine sufficiency and consumption among the women in the first trimester of pregnancy living in areas of the Voronezh region with varying levels of iodine consumption.

**MATERIALS AND METHODS:**

MATERIALS AND METHODS. One hundred women in the first trimester of pregnancy living in areas of the Voronezh region were investigated. All the participants completed a questionnaire regarding the presence of diseases, medication intake, and nutrition. They were examined by an endocrinologist, had their thyroid gland palpated, underwent an ultrasound of the thyroid gland, and provided a single portion of daily urine (before 12:00), followed by the determination of iodine concentration in the urine and calculation of the median ioduria. An analysis of samples of table salt obtained from households was conducted to check for iodine (potassium iodate) using a rapid qualitative analysis method. Blood samples were taken to determine the levels of thyroid-stimulating hormone (TSH), antibodies to thyroid peroxidase (Anti-TPO antibodies), selenium, and zinc in the serum.

**RESULTS:**

RESULTS. The median ioduria was 87.35 (≥150) µg/l. The proportion of households using iodised salt was 23% (n=23). Moderate increased iodine intake was observed in 12% of women; appropriate iodine intake — 15%; and insufficient iodine intake — 73%. Only 23% of women use iodised salt, and 6% regularly take iodine-containing medications, indicating inappropriate iodine consumption and a near-complete absence of iodine prevention in the at-risk group for iodine deficiency diseases. The median TSH level was 1.19 mIU/l, which falls within the reference range (0.4–4 mIU/l). The median selenium concentration was 0.098 µg/ml, which also corresponds to the reference range (0.07–0.12 µg/ml), with no significant differences in selenium concentration found between residents of Voronezh and surrounding areas. According to the results of the ultrasound of the thyroid gland, signs of thyroid pathology (nodular and autoimmune combined) were identified in 55% (n=55) of the examined pregnant women.

**CONCLUSION:**

CONCLUSION. Extremely low levels of iodised salt consumption in households have been identified and Iodine deficiency intake was found in pregnant women.

## АННОТАЦИЯ

В России повсеместно существует риск развития йододефицитных заболеваний (ЙДЗ) из-за недостатка йода в рационе [1][2][3][4]. Недостаточное поступление в организм йода приводит к разворачиванию цепи приспособительных процессов, направленных на поддержание нормального синтеза и секреции гормонов щитовидной железы. Однако, если дефицит йода сохраняется достаточно долго, происходит срыв механизмов адаптации с последующим развитием ЙДЗ [5]. Основные группы риска, в которых последствия недостатка йода в питании наиболее серьезны, — беременные и кормящие женщины, дети от 0 до 3 лет [1][3][4]. Во время беременности потребность в тиреоидных гормонах, а следовательно, в йоде, увеличивается [1][6]. Проживание в регионе легкого йододефицита, где отсутствует адекватная профилактика ЙДЗ, может стать причиной развития зоба у беременных женщин [7][8]. Нарушение функции щитовидной железы (ЩЖ) у беременных женщин, в том числе при дефиците йода тяжелой и средней степени тяжести, может привести к гестозу, хронической внутриутробной гипоксии плода, угрозе прерывания беременности, аномалиям развития плода [9], нарушениями в формировании ЦНС у плода. У детей возникают необратимые дефекты в интеллектуальном развитии в случае недостаточности йода в период внутриутробного развития и в возрасте до 2 лет [10][11].

Целью исследования было оценить йодную обеспеченность женщин в первом триместре беременности, проживающих в районах Воронежской области с различным уровнем потребления йода.

## МАТЕРИАЛЫ И МЕТОДЫ

В течение сентября-октября 2024 г. проводилось обследование 100 женщин в I триместре беременности. С целью охвата городского и сельского населения исследование проводилось в пяти районах Воронежской области: г. Воронеже — 65 женщин, г. Нововоронеже — 10 женщин, Бобровском районе — 10 женщин, Бутурлиновском районе — 10 женщин, Ольховатском районе — 5 женщин.

Исследование было одобрено этическим комитетом ФГБОУ ВО ВГМУ им. Н.Н. Бурденко, все участники прослушали информацию по проблеме и профилактике йододефицита и на добровольной основе подписали информированное согласие.

Обследование беременных включало: сбор анамнеза и анкетирование; осмотр врача-эндокринолога (пальпация ЩЖ, измерение антропометрических показателей (рост, вес)); определение в сыворотке крови уровня ТТГ, антител к тиреопероксидазе (AT-TПO), селена, цинка; определение концентрации йода в моче, исследование образцов пищевой соли из их домохозяйств на наличие в них йода.

Для оценки приверженности населения методам профилактики ЙДЗ все участники исследования заполняли краткий опросник, важными вопросами в котором были: употребление йодированной соли, продуктов, богатых йодом, препаратов йода, а также вопросы о наличии эндокринной патологии и ряда сопутствующих заболеваний и приеме препаратов L-тироксина или тиреостатических препаратов.

Все участницы однократно сдали порцию дневной мочи (до 12:00). Оценка йодурии была выполнена микропланшетным вариантом пробирочного церий-арсенитового метода определения на базе клинико-биохимической лаборатории ГНЦ РФ ФГБУ «НМИЦ эндокринологии» Минздрава России, г. Москва (заведующая лабораторией к.м.н. Л.В. Никанкина, директор чл.-корр. РАН Н.Г. Мокрышева). Оценка адекватности потребления йода беременными, согласно национальными клиническими рекомендациями «Заболевания и состояния, связанные с дефицитом йода», проводилась на основании международных эпидемиологических критериев, рекомендованных BO3: более 500 мкг/л — чрезмерное потребление йода, 250–499 мкг/л — умеренное повышенное потребление йода, 150–249 мкг/л — нормальное потребление йода, менее 150 мкг/л — недостаточное потребление йода [12][13][3][4][6].

Исследование образцов пищевой поваренной соли, полученных из домохозяйств, на наличие йода (йодата калия) осуществлялось экспресс-методом качественного анализа. Принцип метода заключается в изменении окраски раствора крахмала при выделении свободного йода из соли после обработки ее тест-раствором. Степень изменения окраски оценивалась визуально.

Критериями включения в исследование были: подписанное информированное согласие, I триместр беременности, проживание в Воронежской области. Критерии невключения: получение препаратов, которые могут повлиять на результаты исследования (рентгенконтрастные вещества, вводимые при обследованиях за 6 месяцев до исследования, другие препараты, содержащие фармакологические дозы йода выше 1000 мкг), наличие острого заболевания или обострение хронического заболевания.

## Статистическая обработка

Обработка и анализ статистических данных проводились в программах MS Excel 2016 (Microsoft, США), Statзstica 13 (StatSoft, США). Качественные данные представлены в виде абсолютных значений (п) и/или процентов от общего количества — частот (%). В случае описания количественных показателей, имеющих нормальное распределение, полученные данные объединялись в вариационные ряды, в которых проводится расчет средних арифметических величин (М). Совокупности количественных показателей концентрации йода в биологических образцах описывались при помощи значений медианы (Ме) и нижнего и верхнего квартилей (Q1-Q3).

По результатам анкетирования сформирована сводка данных о рационе и употреблении йодированной соли; анализ данных проводился с использованием программы MS Excel.

## РЕЗУЛЬТАТЫ

Исследование с целью оценки йодной обеспеченности включило 100 женщин (18 лет и старше).

Характеристика когорты исследования: средний возраст составил 28,9 года (медианный интервал возраста — 27,5 года).

По данным анамнеза (анкеты обследуемых):

- прием левотироксина по поводу заболевания ЩЖ был у 3% женщин (n=3);

- сопутствующие эндокринные заболевания, нарушения углеводного обмена (включая сахарный диабет) у беременных отсутствовали;

- о наличии вредных факторов труда (токсические производства, ионизирующее облучение) не сообщила ни одна женщина.

По результатам анкетирования, утвердительные ответы о регулярном использовании йодированной соли в питании были получены от 32% (n=32) респондентов. Другие источники йода с пищей (такие как рыба, морепродукты, включая морские водоросли) употребляют с регулярностью 2 и более раза в неделю 47% беременных.

Методом экспресс-анализа качественная реакция на наличие йодата калия была получена в 23% (n=23) из 100 образцов соли, полученных в домохозяйствах беременных. Доля положительных результатов исследования проб соли на содержание йода в разрезе районов Воронежской области представлена на рисунке 1.

**Figure:**
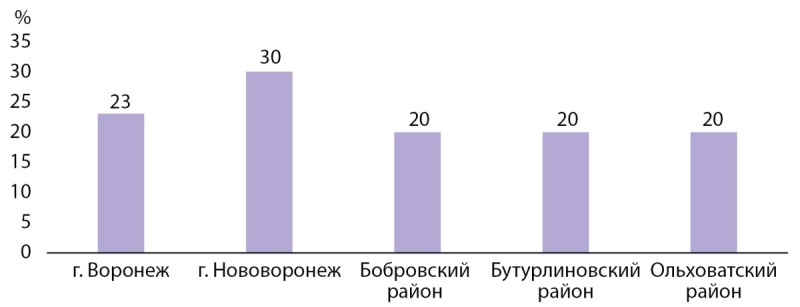
Рисунок 1. Доля положительных результатов исследования проб соли домохозяйств женщин в I триместре беременности на содержание йода в разрезе районов Воронежской области (%).

Для исследования на экскрецию йода с мочой было получено 100 проб от 100 беременных. Медианная концентрация йода в моче (мКЙМ) у беременных составила 87,35 мкг/л в целом по Воронежской области, при этом у беременных жительниц г. Воронеж мКЙМ составила 107,3 мкг/л (n=65) и была значимо выше, чем у жительниц районов области, у которых мКЙМ = 65,9 мкг/л (n=35), что отражено на рисунке 2.

**Figure:**
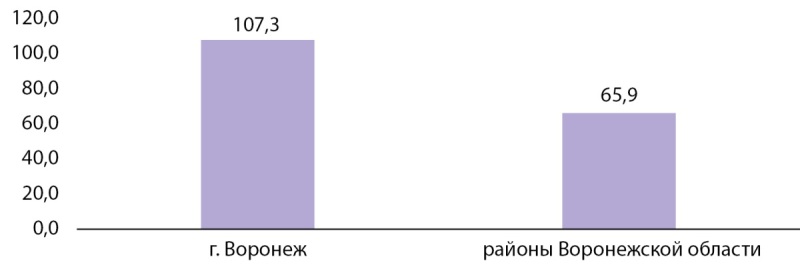
Рисунок 2. Показатели медианы концентрации йода в моче у беременных, мг/л.

Анализ рациона беременных (n=100) показал, что:

- только 32% (n=32) беременных регулярно употребляют йодированную соль, в этой группе беременных мКЙМ составила 146,95 мкг/л, при этом 31% (n=31) беременных регулярно употребляют только йодированную соль (мКЙМ=145 мкг/л), 1% (n=1) беременных регулярно используют йодированную соль и получают профилактику препаратами йода;

- беременные жительницы г. Воронеж, которые регулярно используют йодированную соль в питании, имели более высокий показатель мКЙМ — 142,1 мкг/л (n=27) по сравнению с беременными г. Воронежа, которые не использовали йодированную соль (мКЙМ — 90,5 мкг/л, n=38);

- в группе беременных, не использующих йодированную соль, но регулярно (2 и более раз в неделю) употребляющих морскую рыбу и морепродукты (в т.ч. морские водоросли), мКЙМ составила 74,1 мкг/л (n=21);

- только 6% беременных регулярно получают препараты йодида калия, в этой группе беременных мКЙМ составляет 93,75 мкг/л, при этом 5 из 6 беременных этой группы не употребляют йодированную соль (табл. 1).

**Table:** Таблица 1. Результаты исследования экскреции йода с мочой и анализа рационов питания беременных и кормящих Воронежской области

	мКЙМ у беременных, мкг/л
В целом по Воронежской области (n=100), из них:	87,35
жительницы г. Воронеж, (n=65)	107,3
жительницы районов области, (n=35)	65,9
регулярно употребляют йодированную соль	146,95 (32%)
регулярно получают препараты йодида калия (индивидуальная профилактика)	93,75 (6%)
не употребляют йодированную соль, но регулярно употребляют морскую рыбу и морепродукты и/или продукты, обогащенные йодом	74,1 (21%)

По результатам анализа лабораторных исследований (ТТГ, AT-TПO, цинка и селена) у беременных получены следующие данные:

- медиана уровня ТТГ составила 1,19 мЕд/л, что соответствует референсному интервалу (0,4-4 мЕд/л).

Дополнительно проведен анализ случаев повышения уровня ТТГ более 2,5 мЕд/л. Выявлено 6 беременных:

- у 4 из 6 уровень ТТГ не превышал верхней границы референсного значения, и титр AT-TПO не был повышен (что не рассматривается как нарушение функции ЩЖ), у 2 беременных зафиксирован уровень ТТГ>4 мЕд/л (клиническая ситуация требует уточнения);

- сниженный уровень селена (менее 0,07 мкг/мл) выявлен у 1% обследованных (n=1);

- в целом по области у беременных медианная концентрация селена составила 0,098 мкг/мл, что соответствует референсному интервалу (0,07–0,12 мкг/мл), достоверных различий в концентрации селена среди жителей города Воронежа и районов не установлено;

- сниженный уровень цинка (менее 0,75 мкг/мл) выявлен у 3% обследованных (n=3).

В целом по области у беременных медианная концентрация цинка составила 1,02 мкг/мл, что соответствует референсному интервалу (0,75–1,5 мкг/мл), медианная концентрация цинка среди жителей районов была несколько ниже по сравнению с жителями города Воронежа.

## ОБСУЖДЕНИЯ

ЙДЗ можно предупредить [1][3][4]. В нашей стране накоплен уникальный опыт ликвидации йододефицитных заболеваний, который был перенят многими странами мира [14]. Основными способами профилактики йододефицита является массовое использование йодированной соли и применение препаратов йодида калия среди групп риска развития ЙДЗ [1][3][4][15]. Массовая профилактика йододефицитных заболеваний с помощью йодированной соли — наиболее эффективный метод, рекомендованный ВОЗ, практически не требующий затрат из федерального бюджета [14]. В нашей стране использование йодированной соли было введено в 1999 г. постановлением правительства РФ № 1119 «О мерах по профилактике заболеваний, связанных с дефицитом йода» [1][2].

В начале 1970-x, уже через 10 лет после организации на общегосударственном уровне мероприятий по профилактике ЙДЗ, по результатам проведенных всесоюзных исследований было объявлено, что эндемический зоб как массовое заболевание «ликвидирован или находится на грани ликвидации».

Несмотря на комплекс мероприятий, направленных на реализацию Стратегии повышения качества пищевой продукции в Российской Федерации до 2030 года, утвержденной распоряжением правительства РФ от 29.06.2016 г. №1364, в т.ч. предусматривающей поставки йодированной соли в организации торговли, пищеблоки учреждений здравоохранения, детские сады, школы и др. учреждения, и обязательное использование йодированной соли (ЙС) в питании обучающихся общеобразовательных учреждений (СанПиН 2.4.5.2409-08 с 01.01.2020 г.), в России отсутствует единая государственная политика в сфере профилактике ЙДЗ, охватывающая все население страны, что является главной причиной ежегодного роста заболеваемости патологиями ІЦЖ в РФ.

Результат исследования, проведенного в Воронежской области, показывает, что во всех районах исследования выявлен крайне низкий уровень употребления йодированной соли в домохозяйствах, составляющий 23% при рекомендованном BO3 уровне более 90%.

Результаты анализа рациона питания беременных наглядно демонстрируют низкую приверженность этой когорты к профилактике йодного дефицита путем использования йодированной соли. Также на низком уровне находится употребление йода из других источников пищи (рыба, морепродукты и морские водоросли) — только у 47% беременных.

По результатам исследования экскреции йода с мочой у беременных (всего 100 женщин) в целом для Воронежской области установлено недостаточное потребление йода в данной когорте (мКЙМ у беременных — 87,5 мкг/л, при уровне адекватной обеспеченности >150 мкг/л). Беременные, проживающие в г. Воронеже, имели более высокий показатель экскреции йода с мочой (мКЙМ=107,3 мкг/л, n=65) по сравнению с жительницами районов области (мКЙМ — 65,9 мкг/л, n=45), однако для обеих групп показатель указывает на недостаточную йодную обеспеченность.

Беременные жительницы г. Воронежа, которые регулярно используют йодированную соль, имели наиболее высокий показатель мКЙМ = 146,95 мкг/л (n=32), однако не соответствующий критериям нормальной йодной обеспеченности, по сравнению с беременными г. Воронеж (n =35), которые не использовали йодированную соль в питании (мКЙМ — 88,8 мкг/л).

При анализе рационов беременных установлено, что женщины, которые не употребляют йодированную соль и не получают индивидуальную йодную профилактику препаратами йодида калия, а получают йод только из других источников питания (морская рыба, морепродукты и морские водоросли) имели самые низкие показатели йодной обеспеченности (см. табл. 1).

Настораживает ситуация с практически полным отсутствием йодной профилактики в группах риска развития йододефицитных заболеваний, назначаемой врачами-специалистами согласно действующим Клиническим рекомендациям: только 6% беременных заявили о регулярном приеме йодсодержащих лекарственных препаратов.

## ВЫВОДЫ

За почти 20 лет, на фоне отсутствия системных профилактических мероприятий и крайне низкого уровня использования йодированной соли в домохозяйствах, ситуация с йодной обеспеченностью населения Воронежской области в целом ухудшилась и соответствует легкой степени тяжести йодного дефицита, также недостаточность потребления йода установлена в группах риска у беременных.

## ДОПОЛНИТЕЛЬНАЯ ИНФОРМАЦИЯ

Источники финансирования. Работа выполнена по инициативе авторов без привлечения финансирования.

Конфликт интересов. Авторы заявили об отсутствии потенциального конфликта интересов.

Участие авторов. Все авторы одобрили финальную версию статьи перед публикацией, выразили согласие нести ответственность за все аспекты работы, подразумевающую надлежащее изучение и решение вопросов, связанных с точностью или добросовестностью любой части работы.
